# Ischemic postconditioning protects the heart against ischemia–reperfusion injury via neuronal nitric oxide synthase in the sarcoplasmic reticulum and mitochondria

**DOI:** 10.1038/cddis.2016.108

**Published:** 2016-05-12

**Authors:** L Hu, J Wang, H Zhu, X Wu, L Zhou, Y Song, S Zhu, M Hao, C Liu, Y Fan, Y Wang, Q Li

**Affiliations:** 1 Department of Pharmacology, Jiangsu Provincial Key Lab of Cardiovascular Diseases and Molecular Intervention, Nanjing Medical University, Nanjing, China

## Abstract

As a result of its spatial confinement in cardiomyocytes, neuronal nitric oxide synthase (nNOS) is thought to regulate mitochondrial and sarcoplasmic reticulum (SR) function by maintaining nitroso-redox balance and Ca^2+^ cycling. Thus, we hypothesize that ischemic postconditioning (IPostC) protects hearts against ischemic/reperfusion (I/R) injury through an nNOS-mediated pathway. Isolated mouse hearts were subjected to I/R injury in a Langendorff apparatus, H9C2 cells and primary neonatal rat cardiomyocytes were subjected to hypoxia/reoxygenation (H/R) *in vitro*. IPostC, compared with I/R, decreased infarct size and improved cardiac function, and the selective nNOS inhibitors abolished these effects. IPostC recovered nNOS activity and arginase expression. IPostC also increased AMP kinase (AMPK) phosphorylation and alleviated oxidative stress, and nNOS and AMPK inhibition abolished these effects. IPostC increased nitrotyrosine production in the cytosol but decreased it in mitochondria. Enhanced phospholamban (PLB) phosphorylation, normalized SR function and decreased Ca^2+^ overload were observed following the recovery of nNOS activity, and nNOS inhibition abolished these effects. Similar effects of IPostC were demonstrated in cardiomyocytes *in vitro*. IPostC decreased oxidative stress partially by regulating uncoupled nNOS and the nNOS/AMPK/peroxisome proliferator-activated receptor gamma coactivator 1 alpha/superoxide dismutase axis, and improved SR function through increasing SR Ca^2+^ load. These results suggest that IPostC protected hearts against I/R injury via an nNOS-mediated pathway.

Myocardial injury caused by ischemia/reperfusion (I/R) is characterized by hypercontracture and various forms of cell death in the heart such as necrosis and apoptosis.^1^ Myocardial I/R injury is initiated within the first minutes of reperfusion, and hence this period represents a valuable 'window of opportunity' for myocardial protection.^2^ The most effective strategies are executed in the first minutes of reperfusion, such as ischemic postconditioning (IPostC).^3^

Oxidative stress and Ca^2+^ overload are the major triggers and mechanisms of myocardial I/R injury,^4^ which were caused by increased generation of intracellular reactive oxygen and nitrogen species (ROS/RNS).^5, 6^ IPostC significantly protects cardiomyocytes against I/R injury;^7^ however, the molecular mechanisms remain poorly understood.

Nitric oxide (NO) has an important role in cardiac function. Although NO has emerged as a potent effector molecule for a variety of cardioprotective strategies such as pre- and postconditioning, NO actually has an important role in myocardial I/R injury by acting as a double-edged sword.^8^ NO is most noted for its activation of the classic cGMP-dependent signaling pathway; however, some recent studies have suggested that NO regulates cardiac function by spatial confinement of NO synthase (NOS) isoforms.^9, 10^ For example, endothelial NOS (eNOS) is localized in caveolae where it regulates the l-type Ca^2+^ channel in the plasma membrane, and neuronal NOS (nNOS) is located in the sarcoplasmic reticulum (SR)^11^ and mitochondria (also called mtNOS),^12^ where it regulates SR and mitochondrial function by maintaining Ca^2+^ cycling and nitroso-redox balance.^13, 14^ Given that nNOS regulates intracellular ROS generation and Ca^2+^ levels, which are directly associated with myocardial I/R injury, nNOS may be involved in the pathological condition. Emerging reports have indicated that nNOS overexpression protects mouse hearts from I/R injury^15^ and that nNOS deficiency in mice increases ventricular arrhythmia and mortality after myocardial infarction.^16^

However, whether nNOS is involved in the cardioprotection of IPostC against I/R injury is unknown. We hypothesize that IPostC attenuates myocardial reperfusion injury by reducing oxidative stress and Ca^2+^ overload through an nNOS-mediated pathway.

## Results

### nNOS was involved in the cardioprotection of IPostC against I/R injury

IPostC significantly improved the recovery of left ventricular developed pressure (LVDP) and decreased the elevation of LVEDP in isolated mouse hearts compared with that of the I/R group, which suggests that IPostC improved both systolic and diastolic cardiac function. However, nNOS inhibitors abolished the effect of IPostC on the functional recovery. Unexpectedly, nNOS inhibitors treatment alone during the reperfusion improved the contractile function of the hearts subjected to I/R because it improved the recovery of LVDP at the end of reperfusion (Supplementary Table S1).

**Figure 1:**
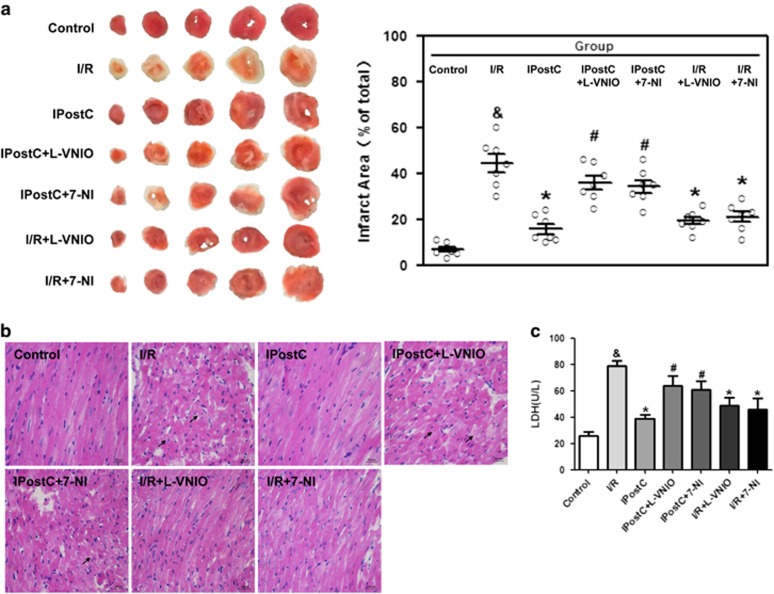
Cardioprotection of IPostC against I/R injury. (**a**) Infarct size was measured at 120 min of reperfusion. IPostC and nNOS inhibitors (l-VNIO and 7-NI) administration alone decreased myocardial infarct size induced by I/R injury, and the effect of IPostC was abolished by nNOS inhibition (*n*=7 per group). (**b**) Representative histological images of hearts at 120 min of reperfusion show a grossly distorted structure, interstitial edema, nuclear vacuolation and frequent contraction band appearance (arrows), which are noted in Figure 2b. In contrast, these structures were largely preserved in IPostC hearts or in hearts treated with nNOS inhibitors during the entire reperfusion (Figure 2b). *n*=3 per group. (**c**) IPostC and nNOS inhibitors (l-VNIO and 7-NI) administration alone decreased LDH levels induced by I/R injury in effluents from isolated mouse hearts. ^&^*P*<0.05 *versus* control; *P<0.05 *versus* I/R; ^#^*P*<0.05 *versus* IPostC

IPostC decreased I/R-induced infarct size (15.7±2.2% *versus* 44.3±3.8%, *P*<0.05). nNOS inhibitor N5-(1-Imino-3-butenyl)-l-ornithine (l-VNIO; 10 *μ*M) blocked the infarct-sparing effect of IPostC (35.9±3.0% *versus* 15.7±2.2%, *P*<0.05), another nNOS inhibitor 7-nitroindazole (7-NI; 10 *μ*M) blocked the infarct-sparing effect of IPostC (34.3±2.8% *versus* 15.7±2.2%, *P*<0.05). Similarly, nNOS inhibitors administration alone, without IPostC, decreased the I/R-induced infarct size (l-VNIO, 19.29±1.66% *versus* 44.3±3.8%, *P*<0.05; 7-NI, 20.86±2.24% *versus* 44.3±3.8%, *P*<0.05) (Figure 1a). In addition, nNOS inhibitor l-VNIO at different doses (5, 10 and 20 *μ*M) were measured, and found that l-VNIO could abolished the infarct-sparing effects of IPostC (Supplementary Figure S5).

Representative histological images of hearts were taken after 120 min of reperfusion. Remarkable ischemic changes such as a grossly distorted structure, interstitial edema, nuclear vacuolation and frequent contraction band appearance (arrows) were noted in the I/R group, whereas normal structures were largely preserved in the IPostC group. nNOS inhibitors l-VNIO and 7-NI abolished IPostC protection, and nNOS inhibitors administration alone also protected the heart structure (Figure 1b).

The lactate dehydrogenase (LDH) level in the I/R group was elevated compared with that of the control group. IPostC decreased LDH levels, and nNOS inhibition abolished this reduction. However, nNOS inhibitors administration alone decreased I/R-induced LDH levels (Figure 1c).

Hypoxic postconditioning (HPostC) increased cell viability and decreased apoptosis in H9C2 cells *in vitro* compared with the hypoxia/reoxygenation (H/R) group. nNOS small interfering RNA (siRNA) abolished the protection of HPostC against H/R injury. However, nNOS siRNA alone during reoxygenation provided cellular protection against H/R injury (Supplementary Figure S2).

These data suggest that nNOS not only mediated IPostC cardioprotection but also may be implicated in myocardial I/R injury when administered alone.

### nNOS expression and activity in isolated heart and H9C2 cells *in vitro*

**Figure 2:**
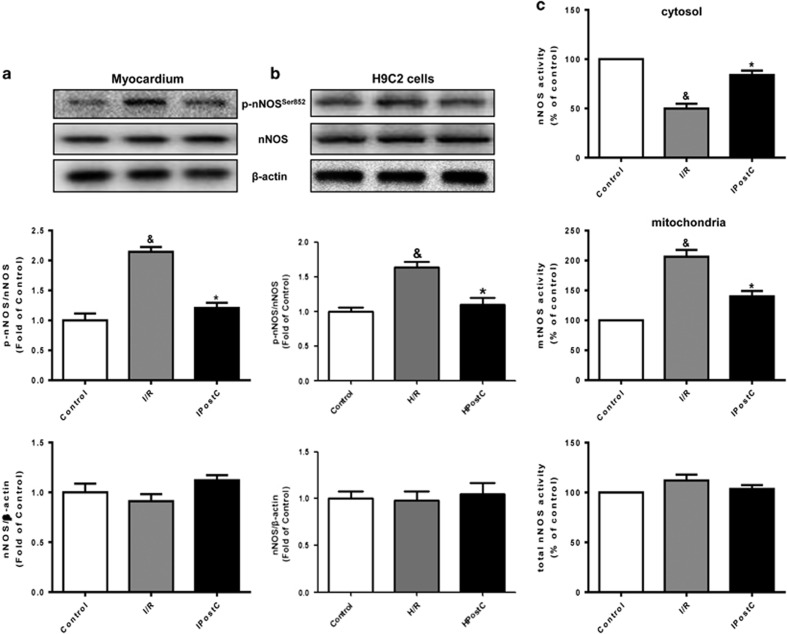
Expression and activity of nNOS in hearts. (**a** and **b**) I/R injury increased the expression of p-nNOS^Ser852^ in the cytosol of the myocardium at 30 min of reperfusion and in H9C2 cells *in vitro* at 30 min of reoxygenation. These effects were decreased by IPostC. (**c**) nNOS activity was decreased in the cytosol but increased in mitochondria of the myocardium at 30 min of reperfusion; these effects were recovered by IPostC (*n*=4 per group). ^&^*P*<0.05 *versus* control; *P<0.05 *versus* I/R

To explore how IPostC affects NOS in hearts, we measured NOS expression. Total nNOS expression in the cytosol (except mitochondria) of myocardial cells was not significantly altered in the I/R group compared with the control and IPostC groups. However, I/R injury markedly increased the expression of p-nNOS^Ser852^ at 30 min of reperfusion, and this expression was decreased in the IPostC group (Figure 2a). A similar result was observed in H9C2 cells cytosol at 30 min of reoxygenation (Figure 2b). As Ser852 in nNOS is an inactive site, these results suggest that nNOS activation was partially suppressed at early reperfusion but that IPostC restored nNOS activity in the cytosol. Moreover, we found that I/R decreased nNOS activity in the cytosol, which was reversed by IPostC. However, I/R markedly increased mtNOS (nNOS) activity in mitochondria, and IPostC recovered mtNOS activity to the level of the control group (Figure 2c).

eNOS expression was also decreased in the I/R group compared with the control group at 30 min of reperfusion, while IPostC restored eNOS content in the myocardium. Inducible NOS (iNOS) expression was not detected in the myocardium at early reperfusion (Supplementary Figure S3).

### IPostC attenuated I/R injury-induced myocardial oxidative stress via nNOS

**Figure 3:**
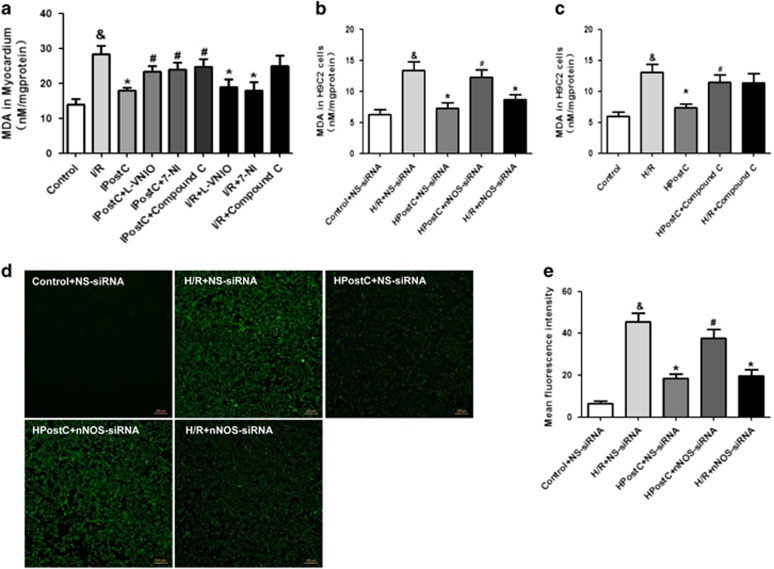
Assessment of oxidative stress in myocardium and H9C2 cells *in vitro*. (**a**–**e**) IPostC decreased MDA and ROS production induced by I/R injury at 30 min of reperfusion; these effects were abolished by nNOS or AMPK inhibition (*n*=4 per group). ROS data were obtained from three independent experiments. ^&^*P*<0.05 *versus* control; *P<0.05 *versus* I/R; ^#^*P*<0.05 *versus* IPostC

To examine whether IPostC protects the heart against I/R injury by attenuating oxidative stress, malonic dialdehyde (MDA) and ROS production was measured (Figure 3). HPostC significantly decreased MDA and ROS production in H9C2 cells compared with the H/R group, and nNOS siRNA abolished the protection of HPostC. Notably, nNOS siRNA alone attenuated the H/R injury-induced generation of MDA and ROS (Figures 3b, d and e). Similar changes in MDA levels were demonstrated in I/R-injured myocardium (Figure 3a). Given that nNOS can generate ROS under defined conditions, in which nNOS is uncoupled to its substrate or cofactors, these data suggest that nNOS uncoupling may occur in the myocardium during early reperfusion.

### IPostC decreased uncoupled nNOS expression in I/R-injured myocardium

**Figure 4:**
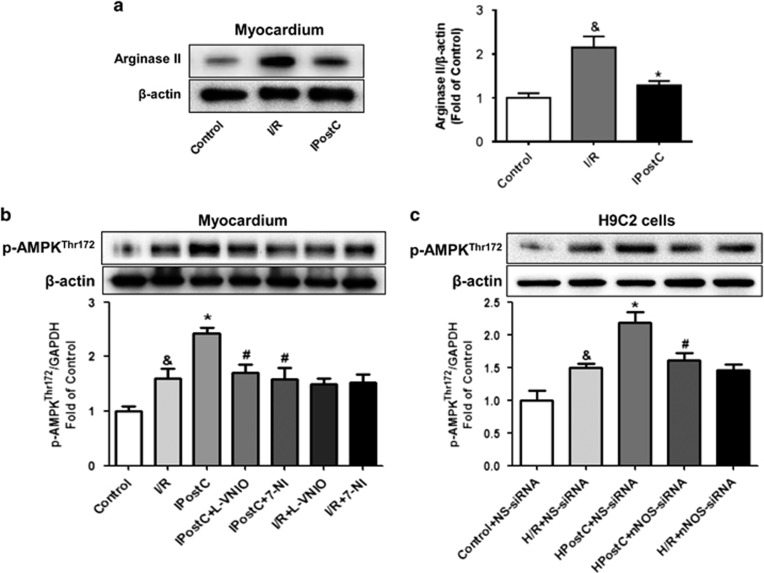
Expression of arginase II, p-AMPK^Thr172^ and *β*-actin in the myocardium at 30 min of reperfusion. (**a**) Arginase II expression was significantly increased in the I/R group; this increase was downregulated by IPostC. (**b** and **c**) p-AMPK^Thr172^ expression was increased in the IPostC group; this increase was abolished by nNOS inhibitors and nNOS siRNA (*n*=3 per group). ^&^*P*<0.05 *versus* control; *P<0.05 *versus* I/R; ^#^*P*<0.05 *versus* IPostC

Arginase is the final enzyme of the urea cycle and competes with nNOS for l-arginine. Depletion of the nNOS substrate l-arginine can result in NOS uncoupling, which subsequently generates ROS. To examine whether nNOS uncoupling occurs in I/R-injured myocardium, arginase expression was detected. As shown in Figure 4a, I/R injury significantly increased arginase expression, and IPostC decreased this effect. These data suggest that I/R injury increased arginase expression, caused nNOS uncoupled, and increased ROS production and that IPostC restored these effects.

### IPostC increased AMPK phosphorylation in I/R-injured myocardium via nNOS

To explore the possibility that IPostC attenuates oxidative stress via an nNOS-mediated pathway, we measured the expression of p-AMPK (Thr172) (Figure 4b). I/R increased AMPK phosphorylation in the myocardium. However, IPostC further enhanced AMPK phosphorylation compared with the I/R group. nNOS inhibition abolished the effect of IPostC. nNOS inhibitors administration alone did not affect AMPK phosphorylation compared with that of the I/R group. Similar changes were observed in H9C2 cells used nNOS siRNA (Figure 4c). These data indicate that IPostC increased AMPK phosphorylation via an nNOS-mediated pathway.

### IPostC protected I/R-injured hearts against oxidative stress via AMPK

As shown in Figures 3a and c, IPostC significantly decreased MDA production in myocardium compared with I/R group. However, the AMPK inhibitor compound C abolished the protection of IPostC. Compound C administration alone did not affect I/R injury-induced production of MDA. In addition, similar changes in MDA levels were demonstrated in H/R-injured H9C2 cells. These results indicated that IPostC attenuated oxidative stress via a nNOS/AMPK pathway against I/R injury.

### IPostC increased PGC-1*α* expression and SOD activity via AMPK

**Figure 5:**
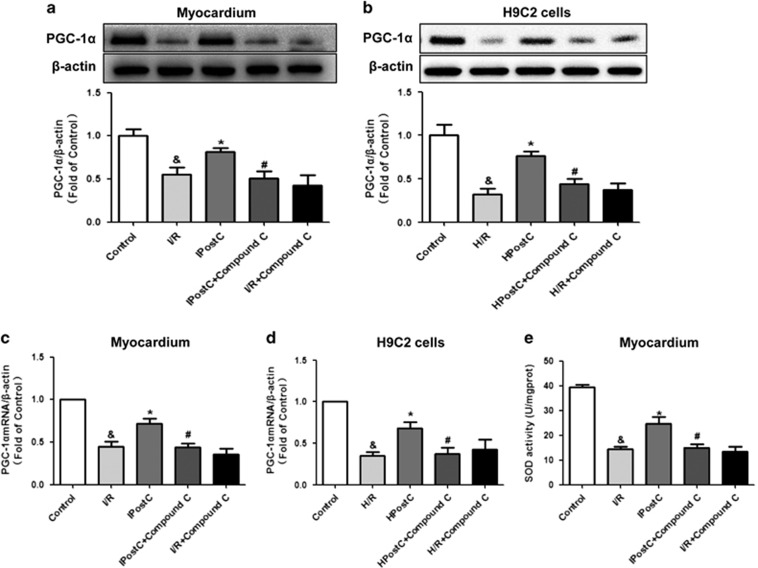
PGC-1*α* expression and SOD activity in the myocardium and H9C2 cells *in vitro*. IPostC increased PGC-1*α* mRNA levels (**c** and **d**) and protein expression (**a** and **b**); these effects were abolished by the AMPK inhibitor compound C. (**e**) IPostC recovered SOD activity, which was abolished by AMPK inhibition (*n*=4 per group). ^&^*P*<0.05 *versus* control; *P<0.05 *versus* I/R; ^#^*P*<0.05 *versus* IPostC

To further explore the mechanism of IPostC protection against oxidative stress via the nNOS/AMPK pathway, we measured peroxisome proliferator-activated receptor gamma coactivator 1 alpha (PGC-1*α*) expression and superoxide dismutase (SOD) activity, which are closely related to oxidative stress (Figure 5). I/R injury decreased the PGC-1*α* mRNA level, which was markedly increased by IPostC, whereas AMPK inhibitor abolished the effect of IPostC. Compound C administration alone did not affect PGC-1*α* mRNA levels in the I/R group (Figure 5a). Similar trends were observed for PGC-1*α* protein expression and SOD activity (Figures 5c and e). Similar results were also observed in H9C2 cells (Figures 5b and d). These data suggest that the AMPK/PGC-1*α*/SOD pathway may have an important role in the cardioprotection of IPostC against oxidative stress.

Considering that IPostC enhanced p-AMPK^thr172^ expression via nNOS in I/R-injured heart, we suggest that IPostC decreased myocardial oxidative stress partially via the nNOS/AMPK/PGC-1*α*/SOD pathway.

### IPostC suppressed myocardial nitrosative stress via nNOS

**Figure 6:**
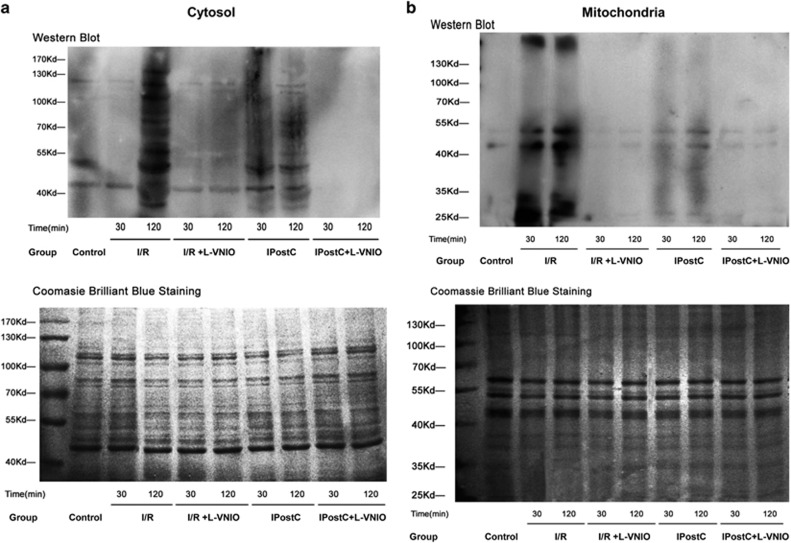
Assessment of nitrosative stress of myocardium exposed to I/R. (**a** and **b**) Nitrotyrosine expression was decreased in the cytosol but increased in mitochondria of myocardium at 30 min of reperfusion; these effects were recovered by IPostC. Nitrotyrosine expression was increased in the cytosol and mitochondria of the myocardium at 120 min of reperfusion; this increase was decreased by IPostC (*n*=4 per group)

NO reacts with O_2_^−^ to form ONOO^−^, which is a major cytotoxic factor implicated in myocardial I/R injury via nitrosative stress.^17^ Nitrotyrosine accumulation, a footprint of ONOO^−^ formation (or more broadly nitrosative stress), in the myocardium was analyzed by western blotting to determine whether IPostC protected the heart against I/R injury by limiting ONOO^−^ generation. As shown in Figure 6, the production of nitrotyrosine was decreased in the cytosol but increased in mitochondria by I/R injury at 30 min of reperfusion. Notably, IPostC returned these effects to physiological levels in the cytosol and mitochondria. Administration of the nNOS inhibitor l-VNIO alone decreased nitrotyrosine formation in I/R-injured myocardium.

These results indicate that ONOO^−^ was formed mainly by nNOS and injured mitochondria during early reperfusion. The injured mitochondria would further contribute to myocardial I/R injury.

### IPostC decreased Ca^2+^ overload and recovered SR function in I/R-injured hearts via nNOS

**Figure 7:**
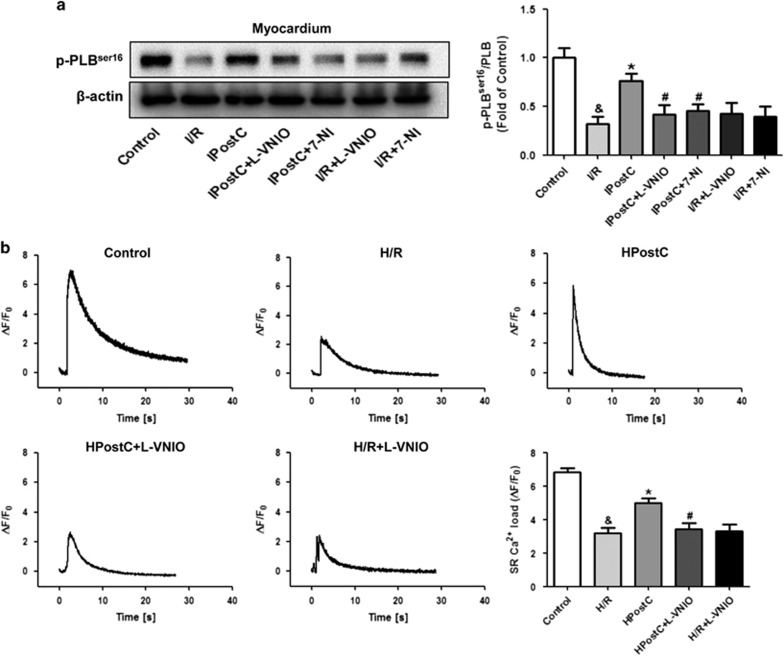
Evaluation of SR function. (**a**) IPostC increased PLB phosphorylation (p-PLB^Ser16^), which was abolished by nNOS inhibition. The data represent samples (*n*=4 per group) taken from myocardium at 30 min of reperfusion. ^&^*P*<0.05 *versus* control; *P<0.05 *versus* I/R; ^#^*P*<0.05 *versus* IPostC. (**b**) Measurements of caffeine-induced Ca^2+^ release from the SR of cardiomyocytes at 30 min of reoxygenation. IPostC increased SR Ca^2+^ load, which was decreased by nNOS inhibition. The data were obtained from three independent experiments. Mean±S.D., ^&^*P*<0.05 *versus* control; *P<0.05 *versus* H/R; ^#^*P*<0.05 *versus* HPostC

nNOS regulates intracellular Ca^2+^ concentrations and cardiac contractility by changing the activities of SR proteins such as sarcoendoplasmic reticulum Ca^2+^-ATPase (*SERCA2a*) and phospholamban (PLB).^18^ Increased PLB phosphorylation enhances *SERCA2a* activity, which may increase the amount of Ca^2+^ that is sequestered in the SR; therefore, p-PLB^Ser16^ expression was determined. Indeed, IPostC increased p-PLB^Ser16^ expression in the myocardium during early reperfusion, and nNOS inhibition abolished this effect (Figure 7a).

Basal Ca^2+^ concentrations at 30 min of reoxygenation were measured in cardiomyocytes *in vitro*. HPostC decreased H/R-induced Ca^2+^ overload, which was consistent with results from previous studies,^19^ and l-VNIO reversed the effect of HPostC (Supplementary Figure S4).

SR Ca^2+^ load was measured using caffeine-induced Ca^2+^ release in cardiomyocytes to elucidate the effect of IPostC on SR function. SR Ca^2+^ load was significantly higher in the HPostC group than in the H/R group, which indicates that HPostC recovered SR function (Figure 7b). However, l-VNIO abolished the effect of HPostC. These data suggest that IPostC recovered SR function by enhancing PLB phosphorylation, which was modulated via an nNOS-mediated pathway.

## Discussion

The primary finding in this study is that IPostC protected isolated mouse hearts against I/R injury partially via an nNOS-mediated pathway. We also suggest that nNOS is one trigger of myocardial I/R injury during early reperfusion. We demonstrated that the selective nNOS inhibitor l-VNIO abolished the cardioprotection of IPostC against I/R injury and unexpectedly decreased myocardial I/R injury to the same extent as IPostC when administered alone during reperfusion.

Whether nNOS has a protective role during acute I/R in the myocardium is controversial.^16, 20, 21, 22, 23^ Our initial studies demonstrate that the selective nNOS inhibitor l-VNIO and 7-NI attenuated the infarct size of isolated mouse hearts and improved cardiac function after I/R. These observations demonstrate that nNOS may have a deleterious role in myocardial I/R injury, particularly during early reperfusion. This result is notable because some researchers have found larger infarct size and higher mortality in nNOS^−/−^ mice than in wild-type (WT) mice.^24^ This discrepancy may be due to the different oxidative stress levels between nNOS^−/−^ mice and WT mice. Cardiac nitroso-redox imbalances are found in nNOS^−/−^^24^ and ob/ob^25^ mice because of decreased nNOS and increased ROS production in the myocardium, which makes nNOS^−/−^ mice more vulnerable to serious injury and mortality after I/R compared with WT mice.

Notably, our results show that I/R injury in isolated mouse hearts is closely associated with nNOS-derived ROS. nNOS inhibitors decrease ROS generation because of the blockade of uncoupled nNOS activity, where O_2_^-^ is formed.^26, 27^ Our data showed that HIF-2*α* was significantly increased by I/R (Supplementary Figure S3D). Some study suggested that HIF-2*α* expression was significantly induced by in heart.^28^ Other study showed that HIF-1*α* and HIF-2*α* protein accumulates after myocardial infarction.^29^ The silencing of HIF-2 but not HIF-1 prevented the activation of arginase II by hypoxia.^30^ Thereby, we hold that arginase is a target of HIF-2*α*. Hypoxia increased HIF-2*α* expression and then upregulated the expression of arginase. In our present study, IPostC significantly decreased arginase expression, increased the nNOS substrate l-arginine and decreased NOS uncoupling, which subsequently decreased ROS and MDA production.

AMPK is a downstream target of mitochondrial ROS generation. Oxidation and subsequent phosphorylation of AMPK are essential for cytoprotection.^31^ Coincidentally, we found that IPostC markedly increased AMPK phosphorylation in I/R-injured hearts. The mitochondrial biogenesis master regulator PGC-1*α* is the major mitochondrial regulator.^32^ Several studies have demonstrated that NO induces mitochondrial biogenesis and upregulates PGC-1*α* expression in different tissues, including the myocardium.^33, 34^ PGC-1*α* overexpression enhanced the expression of antioxidant enzymes including SODs (SOD1, SOD2 and SOD3) and catalase and decreased oxidative stress.^35^ However, these reports did not identify the mechanisms of PGC-1*α* regulation that are mediated by NO-dependent signaling. Notably, our research indicated that IPostC restored nNOS activation in I/R-injured hearts, which increased the concentration and bioavailability of NO. The increased NO can bind to and inhibit cytochrome synthase activity^36^ and creatine kinase activity,^37^ thereby providing a mechanism for NO to increase the AMP-to-ATP ratio within a cell and activate AMPK. This bioactive micromolecule further upregulated the expression of PGC-1*α* mRNA and protein by activating AMPK phosphorylation, subsequently attenuated oxidative stress in I/R-injured hearts. We demonstrate that nNOS-generated NO regulates and oxidative stress via AMPK activation and the subsequent induction of PGC-1*α* and SOD, which may have a key role in IPostC cardioprotection.

IPostC attenuated oxidative injury via the partial recovery of nNOS activity in the SR. As reported previously,^19^ IPostC also limited the generation of ROS, which were derived from the mitochondrial electron transport chain during early reperfusion, and decreased O_2_^-^/NO-derived nitrotyrosine in mitochondria.

Peroxynitrite (OONO^−^) is a RNS that is formed by NO and O_2_^-^ and is a strong cytotoxic compound in many types of heart diseases, including myocardial I/R injury. The NOS inhibitor l-NAME has been reported to protect rat hearts from I/R injury by decreasing OONO^−^ generation.^17^ However, we found that nNOS activity and eNOS expression were decreased and iNOS was not found in the cytosol at 30 min of reperfusion, indicating that NO in the cytosol may be lacking during early reperfusion. In contrast, mtNOS activity was markedly increased at the same time. These results suggest that NO is primarily generated from mtNOS during early reperfusion, which is consistent with some reports that mtNOS-derived NO accounts for >56% of total NO in cardiomyocytes.^38, 39^ Presumably, mtNOS-derived ONOO^−^ is the key agent of oxidative injury in myocardial I/R during early reperfusion.^40^ We found that much more nitrotyrosine was present in the mitochondria than in the cytosol during early reperfusion. ONOO^−^ is known to increase lipid peroxidation and release cytochrome c from isolated mitochondria of rat hearts,^41^ and mtNOS-derived ONOO^−^ induces mitochondrial dysfunction in heart I/R.^42^ Very recently, 10 of 23 proteins were identified from mitochondria as being nitrated by ONOO^−^ after myocardial I/R.^43^ Therefore, mitochondria are major targets of ONOO^−^ nitration. IPostC protected isolated mouse hearts from I/R injury at least partially by reducing mtNOS-derived ONOO^−^, contributing to the suppression of oxygen consumption in the mitochondrial respiratory chain and the consequent cardiac dysfunction and injury.

An abrupt increase in intracellular Ca^2+^ occurs in cardiomyocytes during initial reperfusion.^44^ Intracellular Ca^2+^ overload, secondary to mitochondrial Ca^2+^ overload, induces cardiomyocyte death via the hypercontraction of cardiac cells and mitochondrial permeability transition pore opening, which allows cytochrome c release.^45, 46^ Therefore, SR–mitochondria crosstalk may have an important role in cardiomyocyte survival during I/R. Recently, ONOO^−^ was shown to enhance PLB phosphorylation^47, 48^ and to increase *SERCA2a* activity via S-nitrosylation, which results in SR dysfunction.^49^ Other research has suggested that ROS decrease PLB phosphorylation in the heart.^50, 51^ We found that I/R injury increased nNOS uncoupling, elevated ROS levels and decreased nitrotyrosine production in the cytosol and that these effects were recovered by IPostC via nNOS during early reperfusion. Therefore, we deduced that IPostC raised p-PLB^Ser16^ expression by decreasing ROS levels and recovering nNOS-derived ONOO^−^ to physiological levels in the cytosol, which improved *SERCA2a* activity and subsequently accelerated Ca^2+^ sequestration into the SR to decrease intracellular Ca^2+^ overload and to inhibit hypercontracture. In contrast, IPostC decreased intracellular Ca^2+^ overload, decreased mitochondrial Ca^2+^ concentrations and decreased the activity of Ca^2+^/calmodulin-dependent mtNOS, which subsequently decreased mitochondrial ROS and RNS production and improved mitochondrial function.

In our study, we found that IPostC was protective, and nNOS inhibition abolished IPostC protection. However, nNOS inhibitor administration alone was also actually protective. According to results, high levels of ROS (including ONOO^−^) produced early after I/R injury caused damage to heart. However, in cytoplasm, an appropriate level of ROS is required for PLB phosphorylation, an important regulator of [Ca^2+^]_i_, and an appropriate level of NO in cytoplasm could improve AMPK phosphorylation to reduce oxidative stress. Our data showed that IPostC decreased nNOS activity and ROS level in mitochondria, but increased nNOS activity and ROS level (including ONOO^−^) in cytoplasm to protect I/R-injured heart. nNOS inhibitor administration during IPostC inhibited the increase of nNOS activity, and inhibited the increase of NO and ROS level in cytoplasm, subsequently abolished the effect of IPostC on AMPK and PLB phosphorylation. Thereby, nNOS inhibitor abolished IPostC protection. nNOS inhibitor administration alone abolished the activity of nNOS, which is the major source of ROS, decreased ROS production and had a cardioprotection effect in short time. However, nNOS inhibitor did not increase the activity of nNOS in cytoplasm, this is the difference between IPostC and nNOS inhibitor administration alone, and this is why the cardioprotection of nNOS inhibitor is less effective than IPostC.

**Figure 8:**
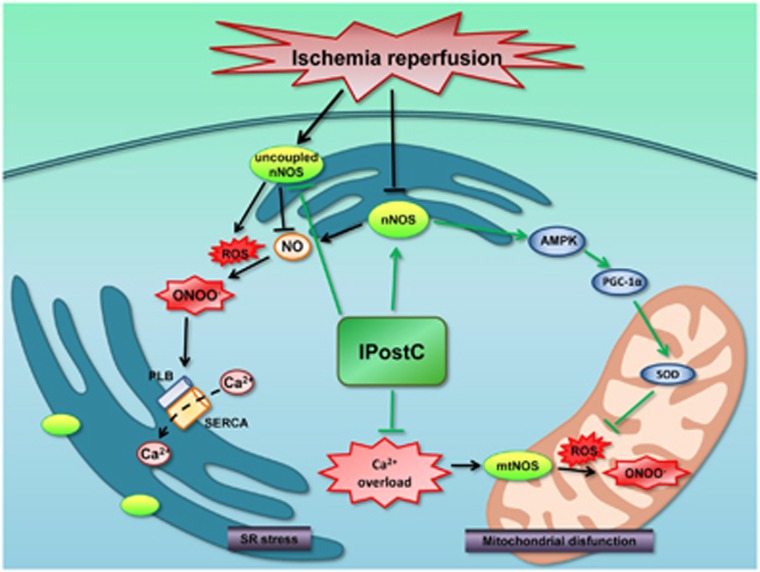
IPostC significantly protects hearts against I/R injury, but its molecular mechanisms remain poorly understood. The hypothesis: IPostC regulates uncoupled nNOS and the nNOS/AMPK/PGC-1*α* axis to decrease oxidative stress and improves SR function by increasing PLB phosphorylation via a nNOS-mediated pathway to decreased intracellular Ca^2+^ overload, which protects hearts against I/R injury. Considering that the effects of nNOS are closely associated with myocardial I/R injury, nNOS may thus be a promising future therapeutic target for ischemic heart disease

This study is the first to demonstrate that an nNOS-mediated pathway has an important role in the cardioprotection of IPostC during early reperfusion. IPostC decreased oxidative stress partially by regulating uncoupled nNOS and the nNOS/AMPK/PGC-1*α*/SOD axis and improved SR function by increasing SR Ca^2+^ load (Figure 8). In addition, owing to its spatial confinement in cardiomyocytes, nNOS has a paradoxical role in I/R injury of isolated mouse hearts, acting as a double-edged sword. The effects of nNOS primarily focus on the SR and mitochondria, which are closely associated with myocardial I/R injury. Therefore, nNOS may be a promising future therapeutic target for ischemic heart disease.

## Materials and Methods

All animal experimental procedures were performed in accordance with the National Institutes of Health Guide for the Care and Use of Laboratory Animals and were approved by Institutional Ethics Committee of Nanjing Medical University.

### Langendorff-isolated perfused heart preparation

The hearts of male C57/B6 mice (weighing 28–30 g, Laboratory Animal Center of Nanjing Medical University, Nanjing, China) were rapidly excised under anesthesia and perfused as described previously.^52^ The LVDP and heart rate were recorded by a MedLab system (Nanjing Medease, Nanjing, China) during a 20-min equilibration period. Mouse hearts were randomly assigned to one of the groups described in Figure 1 and subjected to 30 min of no-flow normothermic global ischemia, followed by 120 min of reperfusion. For details, please see the Supplementary Materials and methods.

The I/R protocol consisted of a 20-min equilibration period, followed by 30 min of global ischemia and 120 min of reperfusion. The protocol for IPostC was identical, except that IPostC was performed at the beginning of reperfusion, which was composed of 10 s of ischemia and 10 s of reperfusion for six cycles. The selective nNOS inhibitor L-VNIO (10 *μ*M; Enzo Life Sciences International, Inc., Plymouth Meeting, PA, USA), 7-NI (10 *μ*M; Sigma, St. Louis, MO, USA) and the AMPK inhibitor compound C (5 *μ*M; Sigma-Aldrich, Gillingham, UK) were administered at the onset of reperfusion for 120 min (Supplementary Figure S1).

### Culture and experimental protocols for cardiomyocytes and H9C2 cells

Neonatal rat cardiomyocytes were isolated by collagenase from 2-day-old S.D. rats (Laboratory Animal Center of Nanjing Medical University) and purified using Percoll gradient centrifugation as described previously.^53^ H9C2 cells and cardiomyocytes were cultured in high glucose Dulbecco's modified Eagle's medium (DMEM) supplemented with 10% fetal bovine serum. H9C2 cells and cardiomyocytes were transferred to an airtight hypoxic chamber maintained at 37 °C with a humidified atmosphere of 100% N_2_ for hypoxic challenges. In the hypoxic chamber, the culture medium was replaced with serum-free, glucose-free DMEM (pH 6.4) that had been saturated with N_2_ gas for 1 h. Normoxic incubation of the cells in serum-free, low-glucose DMEM (1 g/l, pH 7.4) was conducted in a normoxic incubator gassed with 95% air and 5% CO_2_ at 37 °C.^54^ The general experimental protocols used are described below. Control group: cardiomyocytes were incubated in serum-free, low-glucose DMEM in a normoxic incubator during the entire experimental period. H/R group: cardiomyocytes were subjected to 3 h of hypoxia and 6 h of reoxygenation. HPostC group: cardiomyocytes were subjected to HPostC after hypoxia for 3 h. HPostC was induced by exposing the cells to three cycles of 5 min of hypoxia and 5 min of reoxygenation at the beginning of reoxygenation without a change in culture medium. When 5 min of hypoxia, we continuously feeding nitrogen into the culture bottle to replace the air rapidly using a tube. When 5 min of reoxygenation, we continuously feeding gas mixture (95% air and 5% CO_2_) into the culture bottle to replace nitrogen rapidly using a tube. The selective nNOS inhibitor L-VNIO (10 *μ*M), 7-NI (10 *μ*M) and the AMPK inhibitor compound C (5 *μ*M) were administered at the beginning of reoxygenation for 6 h.

### Infarct size measurement

Hearts were removed from the cannula after 30 min of global ischemia and 120 min of reperfusion, weighed, and sliced into 2-mm transverse sections from apex to base. Then, each slice was incubated with 1% triphenyltetrazolium chloride (Sigma) in phosphate-buffered saline (PBS) at 37 °C for 15 min. Each slice was imaged digitally on both sides. The infarct size of each section is expressed as a fraction of the total area of the left ventricle in this isolated I/R model. Computerized area analysis was performed with Image-Pro Plus software (Media Cybernetics, Silver Spring, MD, USA).

### Hematoxylin and eosin staining (HE)

Mouse hearts were quickly removed, fixed in buffered 10% formalin for 24 h and embedded in paraffin. Then, microtome sections (4 *μ*m) were cut and stained with HE.

### Measurement of LDH activity

LDH levels were measured in samples collected from coronary effluents before ischemia and during the first 30 min of reperfusion for all groups using an LDH Cytotoxicity Assay Kit (Nanjing Jiancheng Bioengineering Institute, Nanjing, China). The values are expressed in units per gram of heart wet-weight per liter (U/g/l).

### Evaluation of cell death

Cell viability was assessed by Trypan blue staining, and the apoptotic cells were measured by an Annexin V-FITC apoptosis detection kit. For details, please see the Supplementary Materials and methods.

### Isolation of mitochondria in cardiomyocytes from mouse heart

Isolation of mitochondria was performed by differential ultracentrifugation as previously described.^55, 56^ Briefly, the heart was homogenized with a glass potter in isolation buffer (225 mM mannitol, 75 mM sucrose, 0.5% BSA, 0.5 mM EGTA and 30 mM Tris-HCl, pH 7.4) and then centrifuged twice at 740 × *g* for 5 min to remove nuclei and cellular debris. The supernatant was centrifuged at 9000 × *g* for 10 min to pellet crude mitochondria, which were resuspended in mitochondria resuspending buffer (MRB: 250 mM mannitol, 5 mM Hepes and 0.5 mM EGTA, pH 7.4). Crude mitochondria were further purified through a Percoll medium at 95 000 × *g* for 30 min in the Beckman Coulter Optima L-100XP preparative ultracentrifuge (Beckman Coulter, Inc, CA, USA). Pure mitochondria were washed twice by centrifugation at 6300 × *g* for 10 min and resuspended in MRB.

### Measurement of nNOS activity

The NOS activity assay is based on the biochemical conversion of [3H] l-arginine to [3H] l-citrulline by NOS. Briefly, hearts were homogenized, and the homogenates were centrifuged in a microcentrifuge for 5 min at 4 °C. The supernatant was separated, and the resulting protein concentration was adjusted to 5–10 mg/ml. nNOS activity was measured using a Cayman Chemicals NOS activity assay kit (Cayman Chemical Company, Ann Arbor, MI, USA).

### Immunohistochemical assay of nitrotyrosine

Slides of paraformaldehyde-fixed myocardial tissues were used to detect the formation of nitrotyrosine, a marker of peroxynitrite (ONOO^−^) generation, by a common immunohistochemical approach.^57^ The slides were incubated with mouse monoclonal anti-nitrotyrosine antibody (Cayman Chemical Company) overnight at 4 °C, rinsed twice in PBS, and incubated with biotinylated secondary antibody for 30 min at 37 °C. The sections were stained with streptavidin–biotin complex (SABC) immunohistochemical kit (Boster Biotech, Wuhan, China). Finally, the slides were counterstained with hematoxylin and examined by light microscope.

### Determination of lipid peroxidation in the myocardium

The level of the lipid peroxidation product MDA in left ventricular tissue and in cardiomyocytes was evaluated by measuring thiobarbital-reactive substances levels as previously reported.^58^ Please see the Supplementary Materials and methods for details.

### Intracellular ROS detection

The level of ROS in cardiomyocytes was detected using the fluorescent probe carboxy dichlorodihydrofluorescein (H2DCF-DA; Sigma) based on the manufacturer's instructions. The medium was removed and carefully washed with a PBS after exposure to 3 h of hypoxia and 30 min of reoxygenation. Cardiomyocytes were loaded with 10 *μ*M H_2_DCF-DA in PBS at 37 °C for 15 min. Images were acquired by a fluorescence microscope at 488 nm excitation and 525 nm emission wavelengths at room temperature. Fluorescence intensity was measured using Image-Pro Plus software (Media Cybernetics, Silver Spring, MD, USA).

### Determination of SOD activity in the myocardium

SOD activity (U/mg protein) in the myocardium was estimated by evaluating the rate of inhibition of nucleotide oxidation with an assay kit (Jiancheng Institute of Biotechnology, Nanjing, China).

### Intracellular Ca^2+^ measurement

Intracellular Ca^2+^ ([Ca^2+^]_i_) in isolated cardiomyocytes was measured as fluorescent signals using confocal microscopy as previously described.^59^ For details, please see the Supplementary Materials and methods.

### Western blot

Frozen mouse heart tissue and cardiomyocyte samples were homogenized on ice with an IKA homogenizer, in RIPA lysis buffer (50 mM Tris-HCl (pH 7.5), 150 mM NaCl, 1% NP-40 (vol/vol), 1 mg/ml SDS, 5 mg/ml hyodeoxycholic acid sodium, 1 mM PMSF, 10 mg/ml aprotinin, 10 mg/ml leupeptin and 10 mg/ml pepstatin A). Homogenates were centrifuged at 12 000 × *g* for 15 min, and the supernatant was collected as total cellular protein. Protein concentrations from SR vesicles, mitochondria, and the cytosol and total cellular protein concentrations were determined using a modified Bradford method. Protein samples were transferred onto polyvinylidene difluoride membranes by electroblotting, and membranes were incubated with primary antibodies for phosphor-AMPK (Thr172), AMPK, PGC-1*α* and iNOS (Santa Cruz, Paso Robles, CA, USA), p-nNOS Ser852 (Bioworld, Dublin, OH, USA), nNOS (Zymed, Carlsbad, CA, USA), eNOS (Abcam, Cambridge, MA, USA) and nitrotyrosine at 4 °C overnight. Goat anti-rabbit or anti-mouse HRP-labeled secondary antibodies were incubated at room temperature for 2 h. Immunoreactive bands were detected by enhanced chemiluminescence (Pierce, Rockford, IL, USA) and quantified by Kodak Image Station 4000 MM PRO (Carestream Health Inc., Rochester, NY, USA).

### Real-time quantitative RT-PCR

Total RNAs were prepared using TRIzol reagent (Invitrogen Corporation, Carlsbad, CA, USA) according to the manufacturer's instructions and used for the detection of PGC-1*α* mRNA. PCRs of PGC-1*α* and *β*-actin cDNA (30 cycles of 15 s of melting at 95 °C, 30 s of annealing at 56 °C and 30 s of extension at 72 °C) were performed with Platinum Taq DNA polymerase (Invitrogen Corporation) using the following primers:

Mouse PGC-1*α* forward: 5′-TATGGAGTGACATAGAGTGTGCT-3′,

Mouse PGC-1*α* reverse: 5′-GTCGCTACACCACTTCAATCC-3′

Mouse *β*-actin forward: 5′- ACCTTCTACAATGAGCTGCG-3′,

Mouse *β*-actin reverse: 5′-CTGGATGGCTACGTACATGG-3′

Rat PGC-1*α* forward: 5′- GCACACATCGCAATTCTCCC-3′,

Rat PGC-1*α* reverse: 5′- CTCTCTGCGGTATTCGTCCC -3′

Rat *β*-actin forward: 5′- CTATCGGCAATGAGCGGTTCC-3′,

Rat *β*-actin reverse: 5′- TGTGTTGGCATAGAGGTCTTTACG-3′.

### Knockdown of nNOS using siRNA in H9C2 cells

The nNOS and negative control siRNAs were purchased from Shanghai GenePharma Co., Ltd. (Shanghai, China). The sequences of each siRNA were as follows: nNOS (forward 5′-GCGAACAACUCCCUCAUUATT-3′ and reverse 5′-UAAUGAGGGAGUUGUUCGCTT-3′), and the negative control (forward 5′-UUCUCCGAACGUGUCACGUTT-3′ and reverse 5′-ACGUGACACGUUCGGAGAATT-3′). Cells were plated at a density of 8 × 10^4^ cells per well in six-well plates. After siRNA was preincubated with Oligofectamine in serum-free Opti-MEM medium (Invitrogen Corporation) for 20 min, cells were transfected with nNOS or negative control siRNA oligoduplexes for 6 h, then cells were incubated at 37 °C in a humidified atmosphere of 5% CO_2_.

### Statistical analysis

The data are expressed as the mean±S.E.M., unless otherwise indicated. Statistical significance was assessed by Student's *t-*test or one-way ANOVA followed by Tukey's *post hoc* test where appropriate. All statistics were calculated by Prism GraphPad 5.0 (GraphPad Software Inc., San Diego, CA, USA) An error probability of *P*<0.05 was regarded as significant.

## Abbreviations

NO: nitric oxide
NOS: nitric oxide synthase
nNOS: neuronal nitric oxide synthase
mtNOS: mitochondrial nitric oxide synthase
iNOS: inducible nitric oxide synthase
eNOS: endothelial nitric oxide synthase
I/R: ischemia/reperfusion
H/R: hypoxia/reoxygenation
IPre: ischemic preconditioning
IPostC: ischemic postconditioning
HPostC: hypoxic postconditioning
SR: saroplasmic reticulum
Ca^2+^: calcium ion
LTCC: L-type Ca^2+^ channel
RyR: ryanodine receptor
SERCA: sarcoendoplasmic reticulum
PLB: phospholamban
mPTP: mitochondrial permeability transition pore
ROS: reactive oxygen species
RNS: reactive nitrogen species
ONOO-: peroxynitrite
XOR: xanthine oxidoreductase
MDA: malondialdehyde
LVSP: left ventricular systolic pressure
LVDP: left ventricular developed pressure
LVEDP: left ventricular end-diastolic pressure
L-VNIO: N5-(1-Imino-3-butenyl)-l-ornithine
TTC: triphenyltetrazolium chloride
HE: hematoxylin-eosin
DCFH-DA: 2,7-dichlorofluorescin diacetate
BSA: bovine serum albumin
