# Supplementary material for: Ischemic postconditioning protects the heart against ischemia–reperfusion injury via neuronal nitric oxide synthase in the sarcoplasmic reticulum and mitochondria
Source: Cell Death Dis. 2016 May 12;7(5):e2222–. doi: 10.1038/cddis.2016.108 (PMC4917647; doi:10.1038/cddis.2016.108)
Supplement: Supplementary Information [file cddis2016108x1.doc]

**Ischemic postconditioning protects** **the heart against ischemia-reperfusion injury via neuronal nitric oxide synthase in the sarcoplasmic reticulum and mitochondria**

Liang Hu^1^, Jichang Wang^1,2^, Hongyi Zhu, Xiaowei Wu, Lu Zhou, Yichen Song, Suhua Zhu, Maojuan Hao, Chao Liu, Yue Fan, Yu Wang, Qingping Li*

Department of Pharmacology, Jiangsu Provincial Key Lab of Cardiovascular Diseases and Molecular Intervention, Nanjing Medical University, Nanjing, 210029, P.R.China.

^1^These authors contributed equally to this work.

^2^Present address: Max Delbrück Center for Molecular Medicine, Robert Rössle Strasse 10, 13125 Berlin, Germany

*Corresponding author. Tel.: +86-25-86862883; Fax: 86-25-86868469; E-mail: pharm_qpli[@njmu.edu.cn](mailto:qingpingli@yahoo.com.cn) (Q.P.Li)

**Supplementary material and methods**

**Langendorff isolated perfused heart preparation.** C57/B6 mice (weighing 28-30 g) were heparinized (500U/100g, IP) and anesthetized with 10% chloral hydrate (1ml/100g, IP). Hearts were rapidly excised, washed in ice-cold arresting solution (NaCl 120 mmol/L, KCl 30 mmol/L), cannulated via the aorta, and retrogradely perfused at 37°C and 80 mm Hg with Krebs-Henseleit buffer (mM: NaCl 119, KCl 4.8, KH_2_PO_4_ 1.2, MgSO_4_ 1.2, CaCl_2_ 1.0, glucose 10, and NaHCO_3_ 24.9) bubbled with 95%O_2_/5% CO_2_, as described previously[^1^](#_ENREF_1). A water-filled balloon was inserted into the left ventricle and adjusted to a left ventricular end-diastolic pressure (LVEDP) of 5 to 10 mm Hg. The distal end of the catheter was connected to MedLab system (Nanjing Medease, China) via a pressure transducer. The LVEDP and left ventricular developed pressure (LVDP) (peak systolic minus end-diastolic left ventricular pressure) were measured during the process.

**Evaluation of cell death.** As described previously[^2^](#_ENREF_2), trypan blue staining was used to distinguish viable cells from dead cells by haemocytometer cell counts. Cells that are able to exclude the stain were considered viable, and the percentages of trypan blue-negative cells over total cells were used as an index of viability. Cells were counted within 15 min to minimize variability associated with changes in the ratio of stained/unstained cells over time.

Cell apoptosis was measured by Annexin V-FITC Apoptosis Detection Kit (Bipec Biopharma, MA, USA) according to the manufacturer’s protocol. The cells were analyzed by FACScanTM flow cytometer (BD Biosciences, CA, USA). The percentages of total apoptotic cell were calculated by summing the percentages of cells in early apoptosis (Annexin V-positive but PI-negative) and late apoptosis (Annexin V-positive and PI-positive).

**Determination of lipid peroxidation in the myocardium.** The homogenate was centrifuged at 1,000 g at 4ºC for 10 min after mouse heart tissue (100 mg) (or cardiomyocytes) was washed in PBS and homogenized with the IKA homogenizer (IKA-WERKE, Germany) (15 s × 3) in 1 ml of buffer (mM: NaCl 136, surose 10, Tris-HCl 10, EDTA 1, pH 7.4). The 10% homogenate supernatant was mixed with 15% trichloroacetic acid and 0.375% thiobarbituric acid. 0.01% butylated hydroxytoluene was added to the mixture to prevent autoxidation of the sample, and the mixture was heated at 100°C for 15 min. After being cooled off, the mixture was centrifuged at 1,000 g for 20 min, then the absorbance of the supernatant was measured by a spectrophotometer at 532 nm, and results were expressed as nmol per mg protein.

**Intracellular ROS Detection****.** Cardiomyocytes were loaded with 10 μM H2DCF-DA in PBS at 37°C for 15 min after being exposed to 3 h of hypoxia and 30 min of reoxygenation. Images were acquired by a fluorescence microscope at 488 nm excitation and 525 nm emission wavelengths at room temperature, and fluorescence intensity was measured using Image-Pro® Plus software.

**Statistical analysis.** The data are expressed as the mean ± SEM, unless otherwise indicated. Statistical significance was assessed by Student’s *t-*test or one-way ANOVA followed by Tukey's *post hoc* test where appropriate. All statistics were calculated by GraphPad 5.0. An error probability of P < 0.05 was regarded as significant.

**References**

1 Suzuki, M. *et al.* Cardioprotective effect of diazoxide is mediated by activation of sarcolemmal but not mitochondrial ATP-sensitive potassium channels in mice. *Circulation* **107**, 682-685 (2003).

2 Jiao, J.-D., Garg, V., Yang, B. & Hu, K. Novel functional role of heat shock protein 90 in ATP-sensitive K+ channel-mediated hypoxic preconditioning. *Cardiovasc Res* **77**, 126-133 (2008).

**Supplementary table**

**Table S**1 Cardiac function of the mouse hearts among the groups

| Mouse Hearts | LVDP  (mmHg) | LVDP recovery  (% of Preischemic LVDP) | LVEDP  (mmHg) | Heart rate  (b.p.m) |
| --- | --- | --- | --- | --- |
| I/R (n=10)  Preischemia  Reperfusion (30 min)  Reperfusion (120 min) | 97.3 ± 3.8  45.8 ± 3.9  43.6 ± 3.2 |  | 8.5 ± 1.0  21.0 ± 3.4  25.6 ± 4.6 | 388 ± 12  330 ± 17  295 ± 14 |
|  |  |  |  |  |
|  |  | 46.9 ± 3.0  44.9 ± 3.0 |  |  |
| I/R + L-VNIO (n=8)  Preischemia  Reperfusion (30 min)  Reperfusion (120 min) | 103.1 ± 6.5  42.8 ± 5.5  61.7 ± 4.2 |  | 8.0 ± 0.4  23.3 ± 2.6  15.3 ± 0.9 | 371 ± 4  348 ± 12  324 ± 12 |
|  |  |  |  |  |
|  |  | 41.1 ± 3.7  59.9 ± 1.8* |  |  |
| IPostC (n=8)  Preischemia  Reperfusion (30 min)  Reperfusion (120 min) | 93.8 ± 4.2  48.9 ± 3.1  58.4 ± 3.1 |  | 9.5 ± 0.9  17.8 ± 2.0  15.5 ± 2.0 | 364 ± 6  305 ± 17  315 ± 15 |
|  |  |  |  |  |
|  |  | 52.3 ± 2.9  62.5 ± 2.6* |  |  |
| IPostC + L-VNIO (n=8)  Preischemia  Reperfusion (30 min)  Reperfusion (120 min) | 92.7 ± 4.3  44.9 ± 2.4  40.4 ± 4.5 |  | 8.4 ± 0.8  16.5 ± 1.0  25.1 ± 2.0 | 370 ± 5  309 ± 8  315 ± 14 |
|  |  |  |  |  |
|  |  | 47.3 ± 3.0  41.5 ± 3.4^#^ |  |  |

IPostC improved the recovery of LVDP and depressed the elevation of LVEDP in isolated mouse hearts compared with I/R group. Data are means ± SEM. n indicates number of mouse hearts. I/R = ischemia/reperfusion, IPostC = ischemic postconditioning, L-VNIO = N5-(1-Imino-3-butenyl)-l-ornithine. Reperfusion (30 min) means 30 min of reperfusion. Reperfusion (120 min) means 120 min of reperfusion. *P < 0.05 vs. I/R at 120 min of reperfusion; ^#^P < 0.05 vs. IPostC at 120 min of reperfusion.

**Supplementary figures**


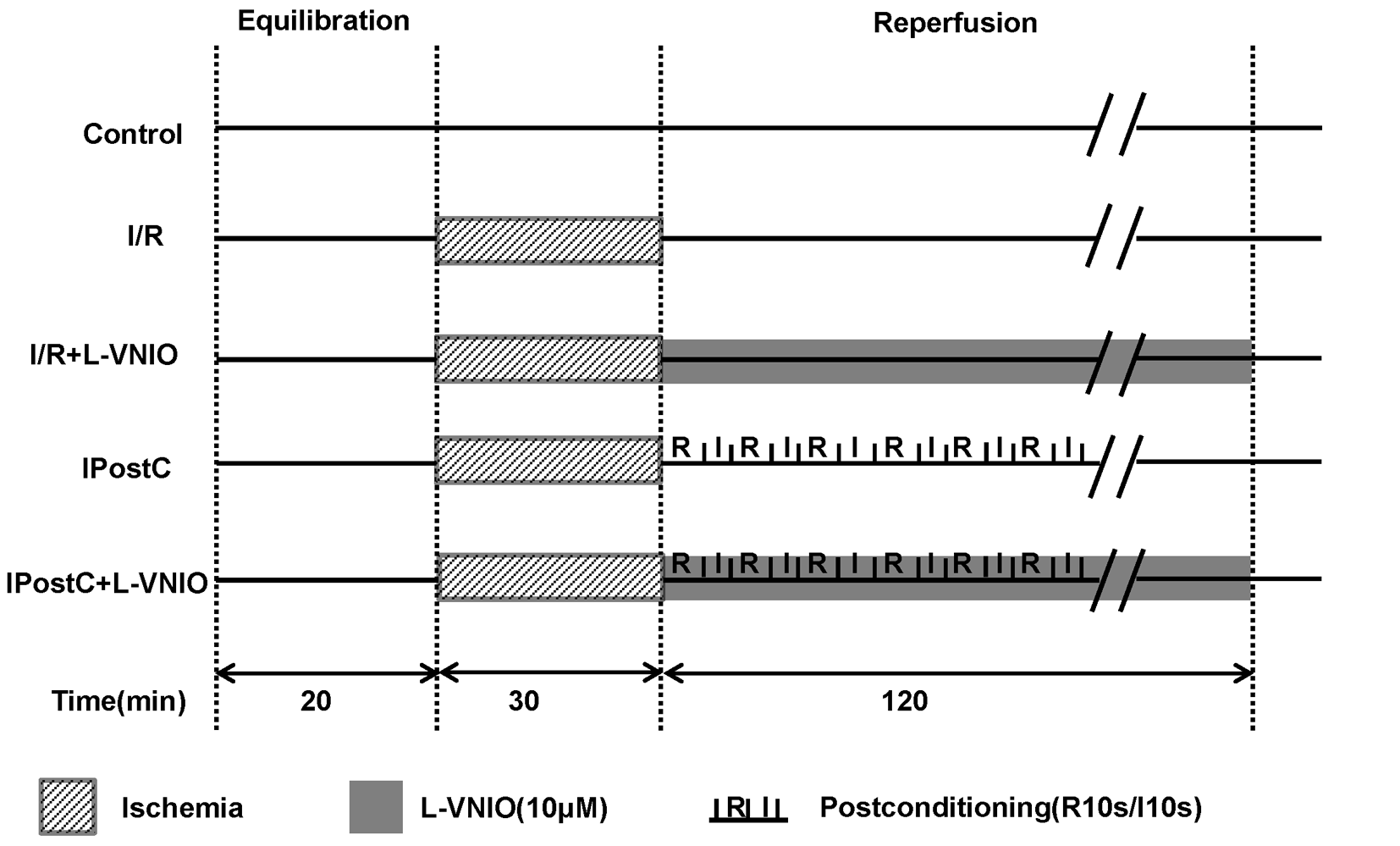


**Figure S1** Protocols for studies of IPostC in isolated mouse hearts. Control = time-matched perfusion of isolated hearts; I/R = unprotected isolated hearts subjected to 30 min of ischemia and 120 min of reperfusion; IPostC = ischemic postconditioning containing 10 s of reperfusion and 10 s of ischemia for six cycles was carried out at the onset of reperfusion after 30 min of ischemia. L-VNIO dissolved in deionized water was administered during the whole reperfusion.


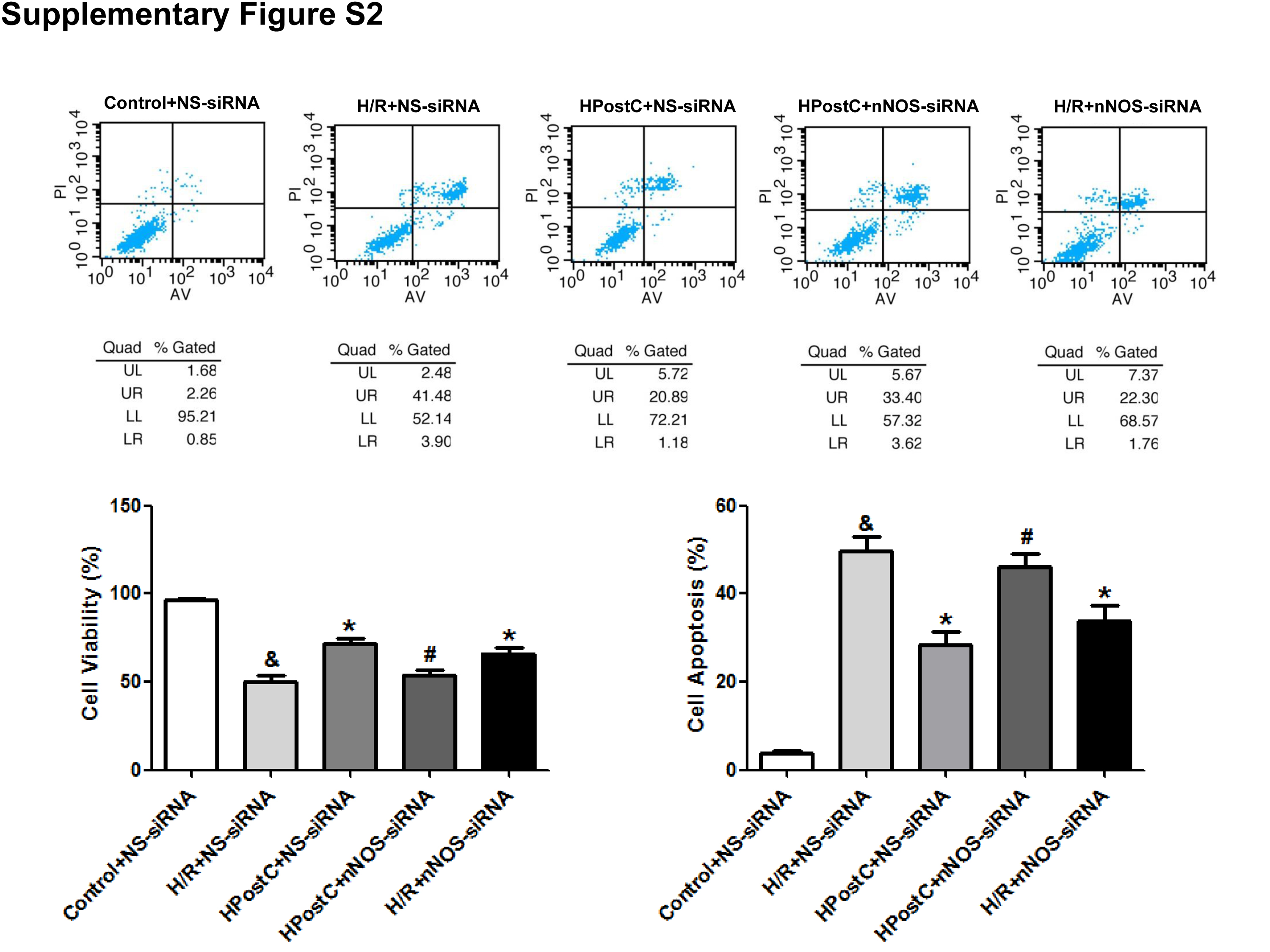


**Figure S2** Cardioprotection of HPostC against H/R injury. Effects of nNOS siRNA, HPostC and HPostC plus nNOS siRNA treatments on cell viability (B) and apoptosis (C) of H9C2 cells subjected to hypoxia/reoxygenation. n = 6 for determination of cell viability; Three individual experiments in each group were performed to determining cell apoptosis. ^&^ P < 0.05 vs. Control; *P < 0.05 vs. H/R; ^#^P < 0.05 vs. HPostC


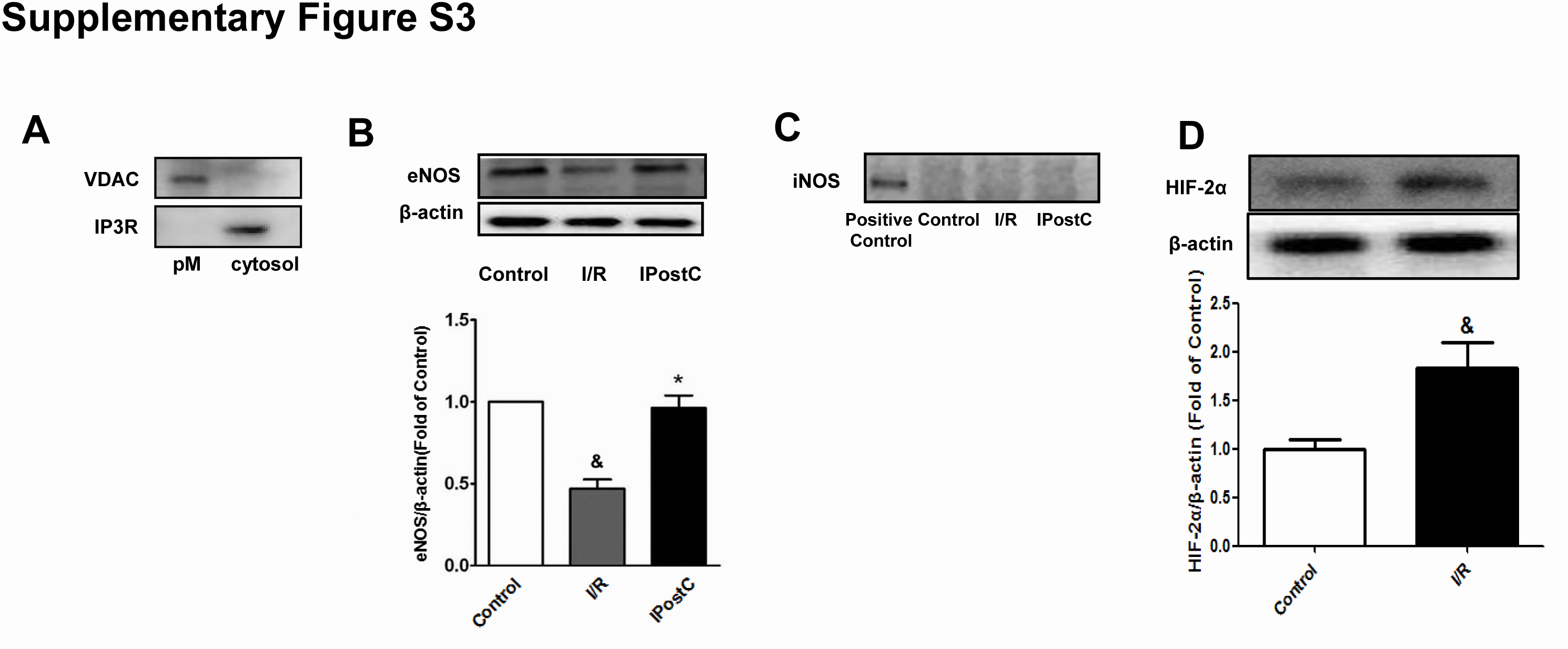


**Figure S3** **(A)** The purity of mitochondria isolated from hearts. There is no sarcoplasmic reticulum (SR) in isolated mitochondria. pM, pure mitochondria. IP3R were used as SR marker, and VDAC was used as mitochondrial marker. (B). IPostC increased eNOS expression compared with I/R group. (C) iNOS expression was not detected in myocardium at the early time of reperfusion. Positive control: cardiomyocytes incubated in hypoxic conditions for 6 h, followed by 12 h of reoxygenation treatment. (D) I/R increased HIF-2α expression compared with Control group. n=3/group. ^&^P < 0.05 vs. Control; *P < 0.05 vs. I/R.

**
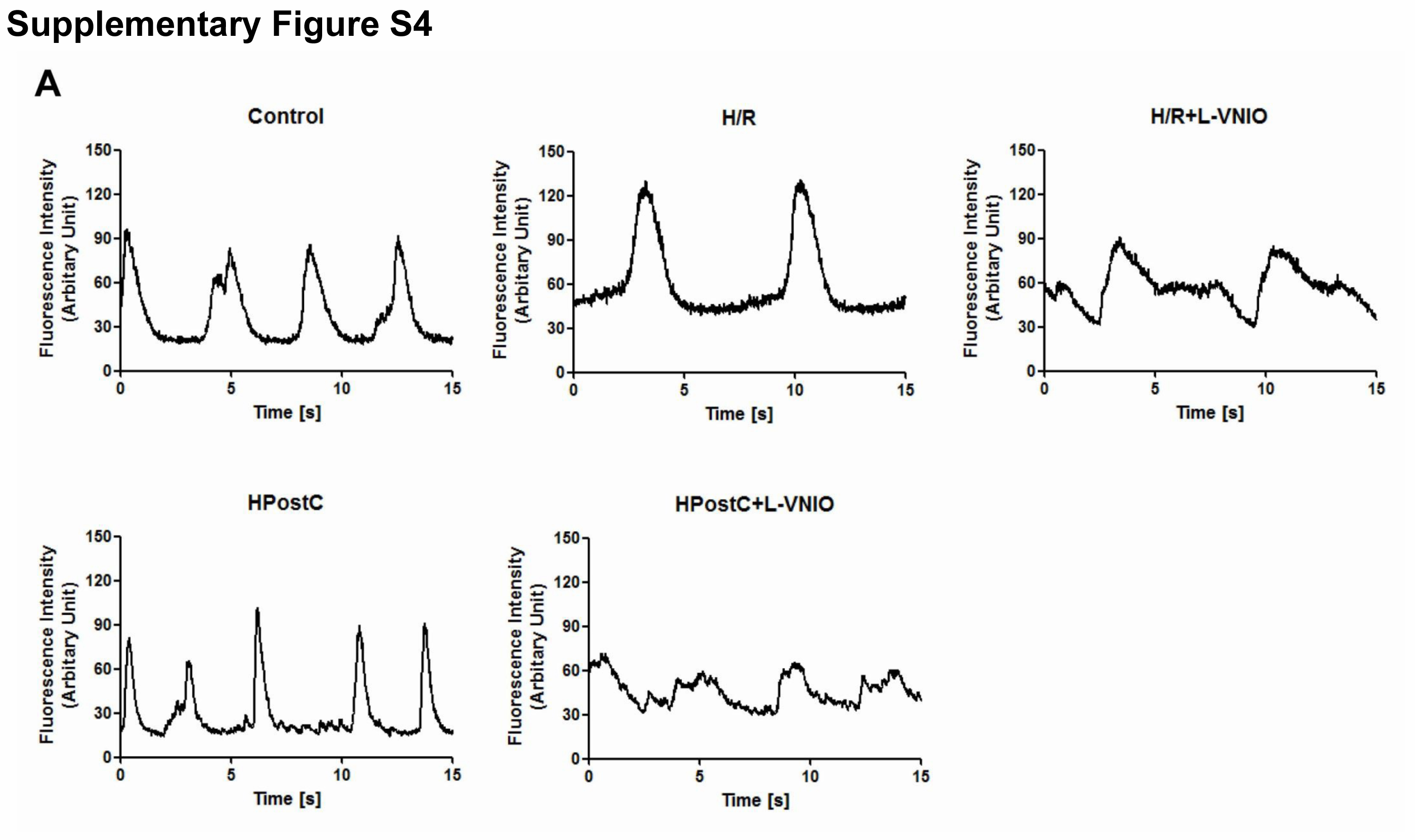
**

**Figure S4** (A) Measurements of intracellulars Ca^2+^ ([Ca^2+^]_i_) at 30 min of reoxygenation. HPostC decreased intracellular Ca^2+^ overload compared with H/R group, which was abolished by nNOS inhibition. Three individual experiments in each group were performed.


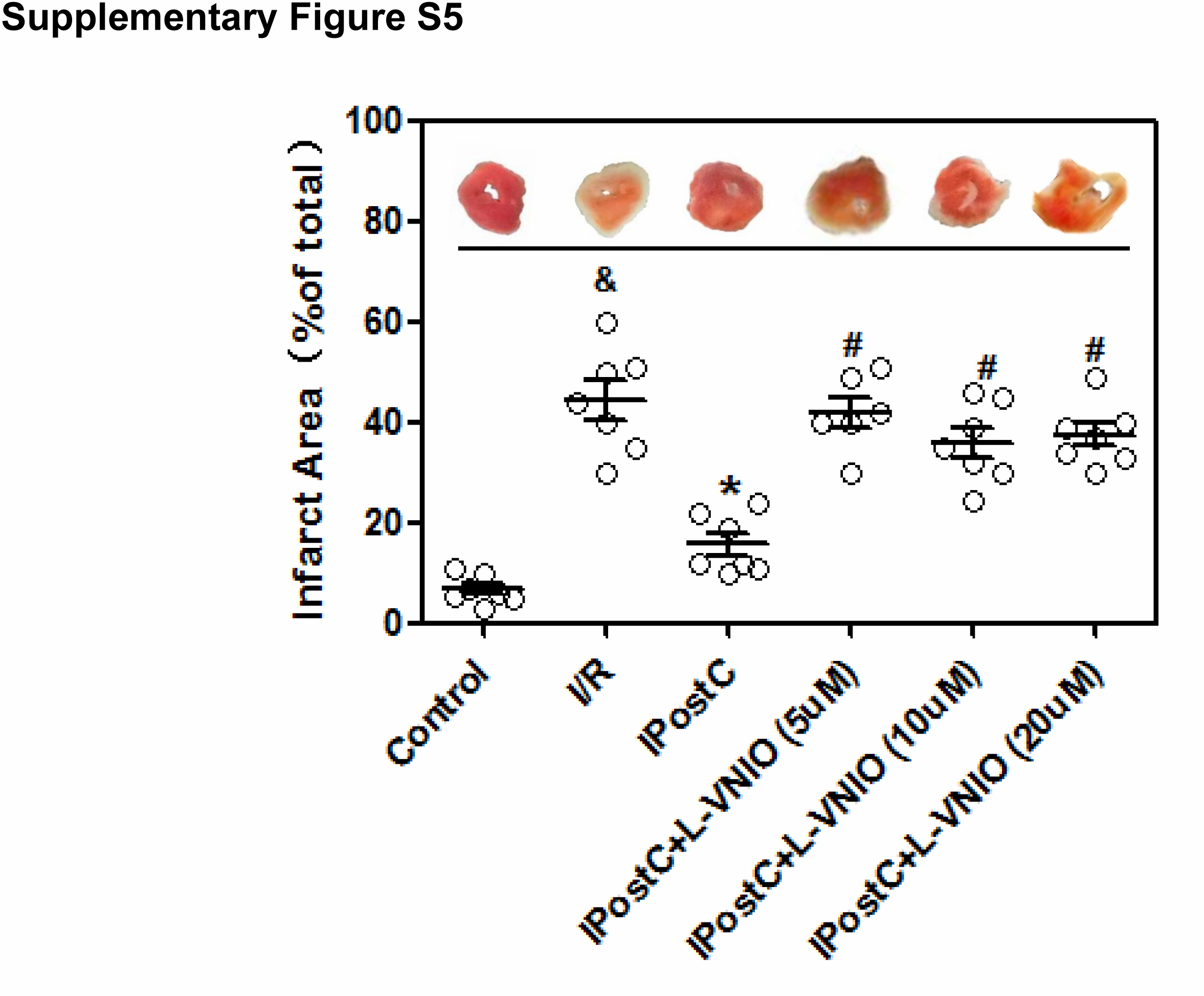


**Figure S5** Infarct size was measured at 120 min of reperfusion. IPostC decreased myocardial infarct size induced by I/R injury, and the effect of IPostC was abolished by nNOS inhibitor L-VNIO at 5 uM, 10 uM and 20 uM. L-VNIO (10 uM) was more effective at these doses. (n = 7/group).
